# A Longitudinal Study of CogEvo’s Prediction of Cognitive Decline in Older Adults

**DOI:** 10.3390/healthcare12141379

**Published:** 2024-07-10

**Authors:** Sadanobu Ichii, Hikaru Oba, Yoshikuni Sugimura, Yichi Yang, Mikio Shoji, Kazushige Ihara

**Affiliations:** 1 Department of Social Medicine, Hirosaki University Graduate School of Medicine, Hirosaki 036-8562, Japan; styyaek_0518@yahoo.co.jp (S.I.); y.sugimura@hirosaki-u.ac.jp (Y.S.); yangy@hirosaki-u.ac.jp (Y.Y.); 2 Graduate School of Health Sciences, Hirosaki University, Hirosaki 036-8564, Japan; hkroba@hirosaki-u.ac.jp; 3 Department of Neurology, Gunma University Graduate School of Medicine, Maebashi 371-8511, Japan; m-shoji@ronenbyo.or.jp

**Keywords:** computer-based screening device, CogEvo, cognitive decline, mild cognitive impairment, dementia

## Abstract

The predictive abilities of computer-based screening devices for early cognitive decline (CD) in older adults have rarely been longitudinally examined. Therefore, this study examined the ability of CogEvo, a short-duration, computer-based cognitive screening device requiring little professional involvement, to predict CD among community-dwelling older adults. We determined whether 119 individuals aged ≥ 65 years living in Japanese rural communities who scored ≥ 24 on the Mini-Mental State Examination (MMSE) at baseline developed CD by annually administering the MMSE to them. CD was defined as an MMSE score of ≤23. At baseline, the overall CogEvo judgment grade, with lower grades indicating better cognitive function, was calculated from the results of various cognitive tasks. Over 2 years, 10 participants developed CD. Participants with grades of 4 had a higher percentage of CD cases than those with grades of ≤3 (*p* < 0.01). This relationship remained significant after controlling for possible confounders, including the MMSE score at baseline. The sensitivity and specificity of the CogEvo grade cutoff of 4 were 50.0% and 93.6%, respectively. In conclusion, CogEvo may be an efficient tool for identifying individuals at a high risk for dementia. The possibility of missing CD cases should be considered when using CogEvo for screening.

## 1. Introduction

The number of people with dementia is rapidly increasing as the older adult population increases. Cognitive impairment in older adults with dementia reduces their independence and impairs their quality of life. It also increases stress on families and caregivers and contributes to the country’s medical costs [1]. Annually, approximately 15% of patients with mild cognitive impairment (MCI) develop dementia [2]. Therefore, preventing the conversion from MCI to dementia may be an important strategy to decrease the morbidity and social burden caused by dementia, and pharmacological and non-pharmacological interventions have been developed for this purpose [3,4]. Early detection of MCI is a prerequisite for the interventions and provides individuals with MCI time and opportunities for voluntary health promotion and developing means to compensate for their functional decline caused by MCI. However, individuals with MCI rarely present to physicians for medical checkups owing to a lack of awareness of MCI among them and their relatives. Therefore, the prevention of dementia may begin with the identification of individuals with early cognitive decline (CD) in the community.

Alzheimer’s disease (AD), which accounts for the majority of dementia cases, clinically involves a gradual decline in memory, language, and other cognitive functions [5]. In AD, pathological changes, such as senile plaques and neurofibrillary tangles, may occur for >10 years before the onset of cognitive symptoms. Pathological changes in the brain progress without symptoms, eventually reaching the dementia stage through preclinical AD and MCI [6].

MCI due to AD has attracted attention as a target for prophylactic treatment with disease-modifying drugs [7]. Amyloid positron emission tomography, cerebrospinal fluid tests, and blood biomarker tests can be used to identify MCI due to AD [8,9]. However, these methods are not cost-effective for population-level screening. In the community, these advanced and expensive tests are substituted with low-cost and non-invasive tests that can identify MCI due to AD [10]. Therefore, these tests should detect CD and neurodegeneration at a mild stage, creating more time for action and reducing the risk of dementia. 

Currently, primary care physicians and other healthcare practitioners use the Mini-Mental State Examination (MMSE) as a population-level screening test [11]. However, the MMSE has been reported to have learning and ceiling effects; therefore, it may not accurately assess CD at very mild stages [12]. As a questionnaire-based tool for assessing mild CD, the Montreal Cognitive Assessment has been reported to identify MCI with a sensitivity of 80.48% and 100% and a specificity of 81.19% and 87% [13,14]. However, these questionnaire-based screening methods might be unsuitable for identifying cognitive impairment in older adults at the population level owing to complications such as measurement bias by examiners and the need for testing by trained staff [15,16].

Recently, tests using touch panels or tablet computers have been increasingly used to assess cognitive function in older adults [17,18]. Computer-based test batteries prevent learning effects by randomly presenting multiple options for the same evaluation item. Furthermore, the state of cognitive function may be evaluated without necessarily having specialized evaluation knowledge or testing skills [19] because the computers lead the examinees to subsequent questions. In addition, computer-based test batteries are useful for efficiently screening large numbers of people at the population level [20]. A systematic review reported that the Cambridge Neuropsychological Test Automated Battery [21], Computer Assessment of Memory and Cognitive Impairment [22], Computer-Administered Neuropsychological Screen for Mild Cognitive Impairment [23], CogState Brief Battery [24], and Mindstreams [25] yielded promising results for identifying MCI and early dementia. However, each of these tests required >40 min to complete. Because older adults are usually not accustomed to using technology, many of them may be dissatisfied with the length of the test. Recently, in Japan, the National Center for Geriatrics and Gerontology’s Functional Assessment Tool (NCGG-FAT) [26], the Computer-Based Cognitive Assessment Tool (Comp-Based-CAT) [27], and CogEvo® (Total Brain Care. Co., Ltd., Kobe, Japan) have been developed. The NCGG-FAT takes >30 min to complete, similar to traditional computer-based cognitive tests. In contrast, the inspection times for the Comp-Based-CAT and CogEvo are shorter.

CogEvo was originally developed to assess and rehabilitate patients with higher brain function disorders. We extended the use of CogEvo to older adults in the community to detect cognitive impairment at an early stage. We also evaluated the concurrent validity of CogEvo and its ability to capture age-related CD [19]. CogEvo has no ceiling effect and can determine milder CD than MMSE. Furthermore, it does not require an expert for scoring because it can automatically calculate test results and present a comprehensive judgment of the participant. These characteristics suggest that CogEvo can be easily deployed in community settings, including community clubs, clinics, and pharmacies; can be used by nurses during home visits to identify individuals with cognitive impairment; and has the potential to be more proactively used by community-dwelling adults than other computer-based cognitive test batteries for older adults.

Nevertheless, no study has examined the predictive validity of CogEvo for assessing cognitive impairment, nor has there been any longitudinal study using CogEvo. Most studies that evaluated cognitive function using computer-based cognitive test batteries other than CogEvo were also cross-sectional, and only a few of them have investigated whether computer-based test batteries can predict CD longitudinally [17]. Demonstrating the predictive validity of CogEvo could boost its potential for identifying individuals who are likely to experience CD.

Therefore, the aim of this study was to examine the ability of CogEvo to predict CD by conducting a 2-year longitudinal study in community clubs for older adults and compare the results with those generated via the MMSE. We also evaluated the screening ability of CogEvo for CD using a receiver operating characteristic (ROC) curve and associated metrics.

## 2. Materials and Methods

### 2.1. Participants

This longitudinal study collected data for over 2 years from 2017 to 2019. The participants included those who participated in community clubs for older adults held at 33 meeting places in Fukaura Town, a rural town with a population of 7346 and an aging rate of 50.7%, located in the southwestern tip of Aomori Prefecture, approximately 700 km north of Tokyo at the northernmost part of the main island in Japan.

**Figure 1:**
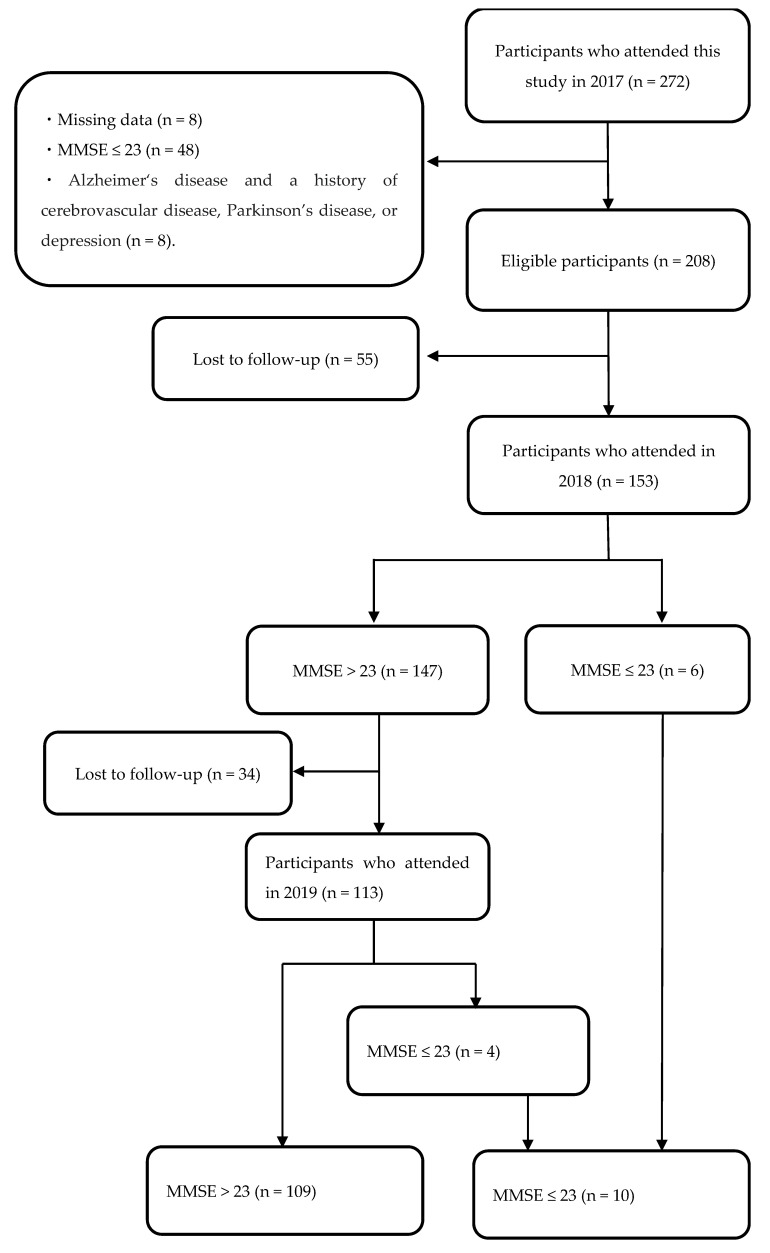
Flowchart of the study participants. Data from 119 participants were used for statistical analysis. MMSE: Mini-Mental State Examination.

Each community club included 2–20 participants. Inclusion criteria encompassed all community club members aged ≥ 65 years with MMSE scores of ≥24 at the baseline survey conducted in 2017, who participated in the baseline survey and follow-up surveys conducted in 2018 and 2019, and who had no missing data. In 2017, 272 community members participated in the baseline survey (Figure 1). Among them, 48 had MMSE scores of ≤23, and 8 had missing data. In addition, eight individuals were excluded for taking medication for AD or having histories of cerebrovascular disease, Parkinson’s disease, or depression. Of the remaining 208 participants from the 2017 baseline survey, 153 participated in the 2018 survey. Among these, 119 participated in the 2019 survey, including those who were identified as having CD in 2018. Ultimately, 119 participants (107 females and 12 males) who completed the three consecutive surveys from 2017 to 2019 were included in the final analysis.

No differences in sociodemographic characteristics and cognitive function at baseline were observed between the 89 individuals who participated in 2017 but could not be followed up with in 2018 or 2019 and the 119 participants who were followed up with until 2019 or until developing CD (Table 1).

### 2.2. Survey Items

In each year, the baseline and follow-up surveys were conducted at a particular date when each community club had its weekly meeting. Pharmacists visited 1 or 2 of the 33 clubs on the same day to conduct a survey and interviewed the participants there to confirm their medical histories and medications. Next, the pharmacists gathered the participants’ sociodemographic information and assessed their MMSE and CogEvo scores (Total Brain Care, Kobe, Japan) in this order. The survey lasted 2 h. Before conducting the surveys, all participants were confirmed to have no vision or hearing issues. This study was approved by the Hirosaki University Ethics Committee (2017-1039), and all participants provided written informed consent.

### 2.3. CogEvo Cognitive Function Balancer

**Figure 2:**
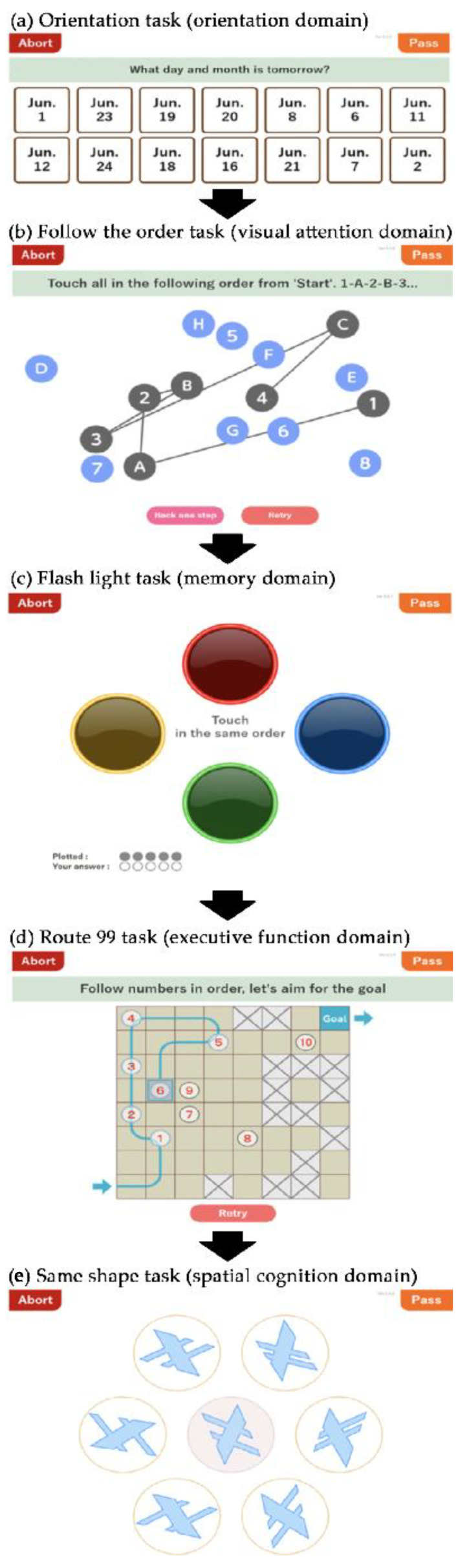
Tasks for the domains of cognitive function assessed with CogEvo. Each panel shows an example from the CogEvo display for each task. (**a**) Orientation task: This task assesses the orientation domain, asking examinees questions such as the date or the day of the week. (**b**) Follow the order task: This task assesses the visual attention domain by instructing the examinee to touch all the numbers and letters on the panel in a specific order. Black lines appear immediately after the examinee touches a number or character. (**c**) Flash light task: This task assesses the memory domain using randomly flashing colored lights. Participants are required to touch the lights in the sequence they memorized. (**d**) Route 99 task: This task assesses the executive function domain by instructing the examinee to move his/her fingers as quickly as possible from a starting point to a goal point within the grid squares. A blue line appears as the examinee traces the squares. (**e**) Same shape task: This task assesses the spatial cognition domain by having the examinee choose the same shape as the one displayed on a swatch from six surrounding pieces.

CogEvo is a test battery that uses a touch panel and was developed for self-checking and training of cognitive function [17]. While CogEvo has 12 types of tasks to examine cognitive function, this study assessed 5 standard tasks to evaluate the domains of “orientation”, “visual attention”, “memory”, “executive function”, and “spatial cognition” [28]. During the CogEvo testing, examiners presented a tablet with CogEvo to each examinee to complete the five tasks that appeared on the touch panel. The testing process started with an “Orientation” task, advanced to a “Follow the order” task, a “Flash light” task, and a “Route 99” task, and concluded with a “Same shape” task (Figure 2). At the beginning of each task, CogEvo inquired of the examinees if they were willing to respond to the task; it began once they answered in the affirmative.

Orientation was assessed through questions regarding the date of examination, the two dates before and after, the day of the week, and the current estimated time. The questions were randomly presented as 14-day choices for the dates, 7 choices for the day of the week, and 14 choices for the time on the touch panel (Figure 2a). Visual attention was examined through an exercise of touching numbers and characters in order and then alternately touching them on the panel in that same order. For example, a participant may touch 1, 2, 3, …, A, I, U, E, …, followed by 1, A, 2, I, 3, U… Each question consisted of alternating combinations of 6 digits, 12 characters, 8 digits, and 8 characters (Figure 2b). The memory assessment involved memorizing randomly flashing lights of one of four colors (red, yellow, green, or blue). Participants were required to touch the lights they had memorized (Figure 2c). Each light flashed for 1 s, and sometimes the same color flashed in series. The task started with 2 flashes, and the light flashed up to 16 times. Consequently, only the accurate response rate was calculated as the score. Execution function was assessed through a task in which participants moved their fingers as quickly as possible from a starting point to a goal point within the grid squares displayed on the touch panel, following the numbers in order as they were shown on the grid squares (Figure 2d). Participants were forbidden to proceed to grid squares with cross marks or pass through a route once it had been taken. The number of grid squares increased from 16 (4 × 4) to 36 (6 × 6) and 64 (8 × 8) in three stages, and the numbers displayed on the grid squares reached 12. A total of three questions were provided. Spatial cognition was evaluated through a task in which participants aimed to choose the same shape as that which was displayed on a swatch in the center of the panel from six other shapes. For each of the four questions, 7 shapes were randomly selected from 38 patterns. The center and answer figures were shown at different positions and angles (Figure 2e).

When considering the specific task details, we noticed that some of CogEvo’s domain names do not align with the conventional psychology terminology. For instance, the “Follow the order” task resembles the Trail-Making “B” Test, which is commonly recognized as an executive function test, yet it represents the visual attention domain in CogEvo. Furthermore, some CogEvo tasks assess only limited elements of their respective domains. For example, the “Route 99” task assesses simple sequencing, which involves executive function but does not cover other components of executive function. Similarly, the “Flash light” task assesses visual–spatial memory but not verbal memory. Nevertheless, we retained the original domain names of CogEvo as designated by the company, as they are an integral part of the product.

CogEvo results can be self-checked by participants after the completion of the five tasks, and the results of the tasks and overall judgment are displayed on six-level scales from “special grade” to “fifth grade”, respectively. A higher grade value indicates a lower cognitive function. A grade of 1 indicates the second-highest level of cognitive function, and a special grade indicates the highest cognitive function. The grade for each task and overall judgment are automatically calculated from the “correct answer rate” and “reaction time”, which are indexed according to the criteria set by age [29].

### 2.4. Statistical Analysis

We used the independent sample Student’s *t*-test or Mann–Whitney U-test for continuous variables and the chi-square test for categorical data to perform the group-wise comparison. The statistical analyses were performed in R 4.3.3 for Windows [30], and the Type 1 error rate was set as α=0.05. Based on the CogEvo grade classification, an ROC curve analysis was performed to predict CD over 2 years, and the area under the ROC curve (AUC), sensitivity, and specificity were calculated using the following equations:

(1) $$Sensitivity=\frac{True\;positives}{True\;positives+False\;negatives}$$

(2) $$Specificity=\frac{True\;negatives}{True\;negatives+False\;positives}$$

(3) $$AUC=\sum_{i=1}^{n-1}\left\{\left(x_{i+1}-x_{i}\right)\frac{y_{i+1}+y_{i}}{2}\right\}$$

where *x* denotes 1 − Specificity, and *y* denotes the sensitivity.

In this study, we defined the CD and cognitive maintenance groups as those with MMSE scores of <23 and >24, respectively. A logistic regression analysis was performed with the presence or absence of an MMSE score of <23 over 2 years as the dependent variable and the CogEvo grade classification as the independent variable. In Model 1, age, sex, years of education, and number of medications taken at baseline were added as covariates. In Model 2, the same covariates as those in Model 1 were included except for age since the CogEvo grade classification method includes age correction. In Model 3, age, sex, years of education, number of medications taken, and MMSE scores at baseline were added as covariates. Furthermore, in Model 4, the same covariates as those in Model 3 were included except for age.

## 3. Results

The sociodemographic characteristics and cognitive functions of 119 participants at the 2017 baseline are shown in Table 1. The mean age of the participants was 79.2 (±5.8) years; females accounted for 89.9%, and participants with <12 years of education accounted for 75.6%. The mean MMSE score was 27.1 (±1.9) points, and 60.5% of participants had a CogEvo grade of 3. Furthermore, the average time required to conduct CogEvo was 551.1 ± 134.5 s at baseline. 

Of the 119 participants, 6 scored ≤ 23 points on the MMSE at the follow-up survey in 2018, and 4 scored ≤ 23 points on the MMSE at the follow-up survey in 2019. In total, 10 participants developed CD over 2 years (Figure 1). However, none of the participants had cerebrovascular disease, Parkinson’s disease, or depression during this period.

**Figure 3:**
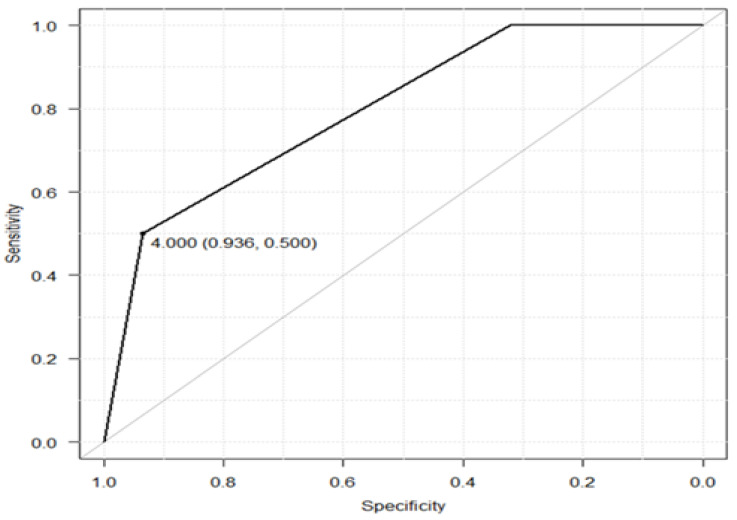
Receiver operating characteristic (ROC) curve for predicting the development of CD (MMSE score ≤ 23) within 2 years after assessment at baseline. The horizontal axis denotes the specificity while the vertical axis denotes the sensitivity. The specificity becomes 0.936 and the sensitivity equals 0.500 when the cutoff value is set to 4. CD: cognitive decline; MMSE: Mini-Mental State Examination.

The AUC showing the relationship between the baseline CogEvo grade classification and 2-year CD was 0.798 (95% confidence interval [CI], 0.682–0.914). According to the Juden method, a CogEvo cutoff value of grade 4 was used as the point at which CD can be predicted, and the sensitivity and specificity were 50.0% and 93.6%, respectively (Figure 3).

The CogEvo grade classification at baseline was significantly related to cumulative CD over 2 years (*p* < 0.01, Table 2). Specifically, the percentage of cumulative incident cases of CD was significantly higher in participants with grades of 4 than in those with grades of 2 and 3. No significant relationship was identified between baseline MMSE scores and 2-year CD (*p* = 0.318). 

Table 3 shows the results of the logistic regression analysis. After adjusting for age, sex, educational history, and number of medications taken, a CogEvo grade of 4 was related to CD over the 2 years (Model 1). Even when age was excluded from the adjustment variables, a CogEvo grade of 4 was related to CD over the 2 years (Model 2).

Even if the MMSE score was added as a covariate, the CogEvo grade of 4 was still related to 2-year CD (Model 3 with age, odds ratio 21.7 [95% CI, 2.7–174.0], *p* = 0.004; Model 4 without age, odds ratio 19.9 [95% CI, 2.75–144.0], *p* = 0.003). 

The results of the logistic regression analysis of the five domains of CogEvo (orientation, visual attention, memory, execution function, and spatial cognition) for CD during the 2 years are shown in Tables S1–S5. The odds ratio of CogEvo memory was 13.6 (95% CI, 1.34–138.0, *p* = 0.027) after controlling for the effect of MMSE with age, sex, educational history, and the number of prescribed medications at baseline (Model 3). The odds ratio, 10.0 (1.17–84.90), remained significant after controlling for the effects of the same variables except for age, as portrayed in Model 4. Furthermore, the odds ratio of CogEvo execution was 2.6 (0.66–10.30) after controlling for the effect of MMSE with sex, educational history, and the number of prescribed medications at baseline (*p* = 0.006, Model 4).

## 4. Discussion

Here, we examined the cognitive function of community-dwelling older adults using CogEvo for 2 years to investigate whether the baseline CogEvo grade was associated with future CD. The percentage of CD cases was higher in participants with CogEvo grades of ≥4 than in those with grades of ≤3 at baseline. The relationship between the CogEvo grade and CD over 2 years was significant after adjusting for the effects of possible confounders. A low sensitivity and a high specificity of the CogEvo grade cutoff were obtained.

In this study, CogEvo predicted CD in older people living in community settings for up to 2 years. The relationship between the CogEvo grade and 2-year CD was significant after controlling for the effects of the MMSE score. Therefore, this relationship was independent of the MMSE assessment results. The percentage of CD cases was not higher in participants with MMSE scores of 24–26 than in those with MMSE scores of ≥27 at baseline. Furthermore, the CogEvo test duration was similar to the duration of the MMSE test [12], although the machine instructions for tasks and automated score calculations are simpler, more consistent, and less prone to examiner bias in CogEvo than in MMSE. CogEvo is a cognitive function test that efficiently identifies and assists individuals who may experience CD in the near future.

This was a longitudinal study. Since the 1980s, many studies have used computer-based test batteries to assess cognitive function. However, most of these were cross-sectional studies, and longitudinal studies are scarce. These test batteries have been categorized based on their primary purposes into equipment designed for evaluation or screening and a separate category reserved for screening equipment with very short durations. The equipment designed for evaluation or screening (the older tests) was typically developed earlier and required a longer time to complete because they focused on a large number of cognitive domains. In addition, the older tests were sometimes used as an adjunct to the treatment of MCI and dementia [31,32]. In contrast, screening equipment with very short testing durations is achieved by targeting fewer domains [33]. Recently, a newer short-duration screening test, the Comp-Based-CAT, was used longitudinally, revealing that cognitive function based on the Comp-Based-CAT at baseline correlated with 2-year CD in community-dwelling older adults, similar to the findings with CogEvo. The Comp-Based-CAT can be completed in 10–15 min, whereas CogEvo requires <10 min. The examinees would likely not be informed of Comp-Based-CAT test results immediately, as testing analysts required time to convert multiple scores for different subscales into a standardized z-score for comparison purposes. However, the CogEvo classification for overall judgment used in this study is automatically calculated and promptly indicated to examinees, as they can self-check their test results. The grade classification of CogEvo also does not burden the participants or examiners. Furthermore, the grade levels for overall judgment allow those around testing participants, such as primary care physicians, other medical professionals, and caregivers, to more easily grasp cognitive function levels based on grades or scores for the five different domains.

CogEvo includes the following five domains, which are assessed using computer-based cognitive function tests: “orientation”, “visual attention”, “memory”, “executive function”, and “spatial cognition” [19,29]. Most studies that have used computer-based cognitive function tests reported a relationship between only a single domain of a test and longitudinal cognitive changes, regardless of whether the test assesses multiple domains [34]. As early symptoms of cognitive impairment appear in multiple domains of cognitive function, assessing CD may be difficult in a single domain [35]. Our study found that impairment in two domains of CogEvo, memory and executive function at baseline, could predict future CD (Tables S1–S5). However, the odds ratio for overall judgment was higher than those of the two domains. Grade classification for overall judgment may predict future CD better than the individual domains. Rather than using individual domain evaluations, CogEvo’s overall judgment results may convey the need for early support for patients and those around them.

The AUC for the relationship between the baseline CogEvo grade and 2-year CD was 0.798. In addition, the sensitivity and specificity of the CogEvo grade cutoff of 4 were 50.0% and 93.6%, respectively. The low sensitivity indicates that CD may appear in those who had good baseline CogEvo grades (≤3). When using CogEvo for screening, people who require early support may be excluded from the test-positive category. A previous study investigating the screening accuracy of CogEvo for detecting CD showed a sensitivity of 70% and specificity of 60% [19]. The definition of CD in the previous study was an MMSE score of ≤23, which is similar to its definition in our study; however, the way in which cognitive function was evaluated by CogEvo in the previous study differed. Therefore, the findings of the previous studies and those of ours are not directly comparable. The lower sensitivity in our study than that in previous studies does not necessarily mean that CogEvo is more likely to miss cases of CD than other measures since the low sensitivity may reflect the difference between longitudinal and cross-sectional studies. Takahashi et al. [36] reported an AUC of 0.79, sensitivity of 0.76, and specificity of 0.75 for six tasks of the Comp-Based-CAT, a short-running computer-based cognitive function test, to predict the onset of MCI over 2 years. The AUCs of CogEvo and Comp-Based-CAT were almost equivalent, but their sensitivities and specificities differed. In addition, the participants in the Comp-Based-CAT study were older adults in Tokyo, the capital of Japan, whereas CogEvo was used for older adults living in depopulated rural areas. Each of these computer-based cognitive tests has different origins and contexts of development and cannot be compared based on sensitivity and specificity. Therefore, to implement the Comp-Based-CAT in rural older populations, sensitivity and specificity should be examined. CogEvo can be performed in locations that resemble daily life, such as community clubs and homes, as long as the internet is available and its use is not confined to medical institutions such as clinics. The overall judgment of cognitive function for each participant is automatically provided, and psychologists are not required to calculate scores. CogEvo can be used and evaluated by healthcare professionals and care workers. Therefore, in areas where primary care physicians and other health professionals are lacking and healthcare is unevenly distributed, computer-based self-test batteries such as CogEvo can be highly useful.

This study had some limitations. First, it was a field study conducted in only one region. Owing to regional bias, these results may not be generalizable to other populations. Second, the measurement environment was inconsistent. For example, examinees and examiners in some meeting halls experienced noise disruption when conducting the CogEvo test, as they had to share a room with other members of a community club who were carrying out their activities. Third, a measurement bias might occur for CogEvo because the same question as the MMSE, date or day of the week, appeared on the “Orientation” task of CogEvo at approximately 40% chance. It might lead to a better score of orientation than the actual one, resulting in a better CogEvo subclassification grade and potentially leading to a lower sensitivity or a higher specificity. Fourth, an MMSE score of ≤23 was used to define CD, which may oversimplify the classification process and lead to misclassification. Unfortunately, besides MMSE, we did not assess other evaluation items, such as CDR and other effects on daily life. In addition, the number of people with CD over the 2 years was as low as 10, possibly leading to a greater error. Fifth, this study included fewer males than females; therefore, we should be careful to generalize the results to both sexes. Nevertheless, in a survey of local residents, the proportion of female participants tends to be higher than that of males, reflecting the longer life expectancy of females. Sixth, we should note that some of CogEvo’s domain names do not align with the conventional psychological terminology. However, changing them is not feasible as they are integral to CogEvo, a commercial product that has been adopted as a cognitive function test in the Japan-Multimodal Intervention Trial for Prevention of Dementia [37], part of the World-Wide FINGERS Network [38]. Furthermore, as some CogEvo tasks assess only limited elements of their respective domains, CogEvo might not appropriately assess cognitive function. Although adding one to two more tasks other than the standard five tasks of CogEvo may improve its screening accuracy, the addition would increase the examination time of CogEvo and might undermine CogEvo’s advantage of a shorter examination time compared with other cognitive function tests. Finally, the 24-month interval between the baseline and final assessments may not have been sufficient to predict CD. Therefore, in the future, we hope to collect longitudinal data over longer periods.

## 5. Conclusions

Currently, many computer-based cognitive function tests are available. Tests that easily evaluate cognitive function are long-awaited tools to help people with CD in the community. This is the first study that longitudinally examined the ability of CogEvo, which has a short testing duration and little involvement of experts, to predict CD in community-dwelling older adults. A CogEvo grade of ≥4 was associated with a 2-year CD development, whereas the MMSE score was not associated with CD. The level of the AUC, in relation to baseline CogEvo and 2-year CD, was fair, with a relatively low sensitivity and high specificity. CogEvo has been shown to have predictive validity for 2-year CD and may be an efficient tool for identifying individuals at a high risk of dementia; however, the possibility of missing cases of CD should be considered when using CogEvo for screening.

## Figures and Tables

**Table 1:** Sociodemographic characteristics, number of prescribed medications, and cognitive function at baseline.

Participant Number	Study Participants (n = 119)				Withdrawals (n = 89)				*p*-Values
	Number (%)				Number (%)				
Age									
<69 years	8	(	6.7	)	11	(	12.4	)	0.08
70–79 years	53	(	44.5	)	41	(	46.1	)	
80–89 years	54	(	45.4	)	35	(	39.3	)	
90–99 years	4	(	3.4	)	2	(	0.2	)	
Sex									
Female	107	(	89.9	)	80	(	89.9	)	0.995
Male	12	(	10.1	)	9	(	10.1	)	
Years of education									
≥12 years	29	(	24.4	)	29	(	33	)	0.193
<12 years	90	(	75.6	)	60	(	67	)	
Prescription									
≥6	48	(	40	)	34	(	38	)	0.757
<6	71	(	60	)	55	(	62	)	
CogEvo subclassification grade									
Grade 1	0	(	0	)	3	(	0.3	)	0.817
Grade 2	35	(	29.4	)	27	(	30.3	)	
Grade 3	72	(	60.5	)	40	(	44.9	)	
Grade 4	12	(	10.1	)	19	(	21.3	)	
Grade 5	0	(	0	)	0	(	0	)	
CogEvo subclassification grade *	2.8	±	0.6		2.8	±	0.8		0.71
CogEvo Score **									
Total	1084	(	927, 1225	)	1058	(	779, 1261.5	)	0.365
Orientation	274	(	218, 317.5	)	259	(	210.25, 315.5	)	0.493
Follow the order	172	(	152.5, 192.5	)	167.5	(	117.25, 192.75	)	0.388
Flash light	290	(	190, 350	)	265	(	150, 340	)	0.642
Route 99	150	(	116.5, 150	)	150	(	103.75, 150	)	0.531
Same shape	242	(	170, 292	)	223	(	163, 290.75	)	0.541
CogEvo examination time (s) **	550.33	(	478.84, 636.55	)	559.61	(	464.39, 654.27	)	0.868
MMSE **	27	(	26, 29	)	26	(	25, 29	)	0.148

*, Mean ± SD. **, Median (IQR). *T*-test or U-test for continuous variables. Chi-square test for categorical variables. MMSE, Mini-Mental State Examination; SD, standard deviation; IQR, interquartile range.

**Table 2:** Relationship between baseline cognitive function and cognitive decline over 2 years.

	MMSE > 23				MMSE ≤ 23				*p*-Values
	n	(	%	)	n	(	%	)	
CogEvo subclassification at baseline									
Grades 2 and 3	102	(	95.3	)	5	(	4.7	)	<0.001
Grade 4	7	(	58.3	)	5	(	41.7	)	
MMSE at baseline									
≤27	65	(	94.2	)	4	(	5.8	)	0.318
24–26	44	(	88.0	)	6	(	12.0	)	

MMSE: Mini-Mental State Examination. Chi-square test was applied.

**Table 3:** Odds ratios of CogEvo subclassifications at baseline for cognitive decline over 2 years.

	Variables	OR	95% CI					*p*-Values
Model 1	Age	1.24	(	1.03	–	1.49	)	0.023
	Sex	1.44	(	0.13	–	16.10	)	0.766
	Education	0.86	(	0.08	–	9.69	)	0.901
	Prescription	3.74	(	0.47	–	30.10	)	0.214
	CogEvo subclassification grade 2 and 3/4	26.1	(	3.51	–	193.00	)	0.001
Model 2	Sex	1.91	(	0.19	–	19.60	)	0.588
	Education	1.87	(	0.19	–	18.20	)	0.590
	Prescription	7.35	(	0.98	–	55.10	)	0.523
	CogEvo subclassification grade 2 and 3/4	27.4	(	4.10	–	182.00	)	0.001
Model 3	Age	1.23	(	1.02	–	1.48	)	0.027
	Sex	1.43	(	0.13	–	16.30	)	0.773
	Education	0.88	(	0.08	–	10.20	)	0.916
	Prescription	4.03	(	0.51	–	32.10	)	0.188
	CogEvo subclassification grade 2 and 3/4	21.7	(	2.70	–	174.00	)	0.004
	MMSE *	0.87	(	0.53	–	1.42	)	0.570
Model 4	Sex	2.01	(	0.19	–	21.10	)	0.559
	Education	2.07	(	0.21	–	20.80	)	0.536
	Prescription	7.69	(	1.03	–	57.20	)	0.047
	CogEvo subclassification grade 2 and 3/4	19.9	(	2.75	–	144.00	)	0.003
	MMSE *	0.81	(	0.51	–	1.29	)	0.379

MMSE, Mini-Mental State Examination; OR, odds ratio; CI, confidence interval. * MMSE is a continuous quantity analysis. Logistic regression analysis: Covariates; Age, Sex, Education, Prescription, CogEvo subclassification grade 2 and 3/4, and MMSE.

## Data Availability

Data are available from S.I. with the permission of the Hirosaki University Ethics Committee.

## Supplementary Materials

The following supporting information can be downloaded at: https://www.mdpi.com/article/10.3390/healthcare12141379/s1, Table S1: Odds ratios of CogEvo subclassification for Orientation at baseline for cognitive decline over 2 years; Table S2: Odds ratios of CogEvo subclassification for Follow the order at baseline for cognitive decline over 2 years; Table S3: Odds ratios of CogEvo subclassification for Flash light at baseline for cognitive decline over 2 years; Table S4: Odds ratios of CogEvo subclassification for Route 99 at baseline for cognitive decline over 2 years; Table S5: Odds ratios of CogEvo subclassification for Same shape at baseline for cognitive decline over 2 years.

## Abbreviations

AD: Alzheimer’s Disease; AUC: Area Under the Curve; CD: Cognitive Decline; Comp-Based-CAT: Computer-Based Cognitive Assessment Tool; MMSE: Mini-Mental State Examination; MCI: Mild Cognitive Impairment; NCGG-FAT: National Center for Geriatrics and Gerontology’s Functional Assessment Tool; ROC: Receiver Operating Characteristic.
